# 72 The Impact of Rural Living on Burn Survivors’ Follow-Up Attendance

**DOI:** 10.1093/jbcr/iraf019.072

**Published:** 2025-04-01

**Authors:** Alexa Smith, Mustafa Jundi, Alec McLeod, Garrett Hagwood, Jason Heard, Soman Sen, Tina Palmieri, Kathleen Romanowski

**Affiliations:** University of California Davis; University of California Davis School of Medicine; University of South Dakota; Sutter Roseville Medical Center; University of California Davis; University of California, Davis and Shriners Children’s; University of California, Davis and Shriners Children’s; University of California, Davis and Shriners Children’s

## Abstract

**Introduction:**

Due to the risks of scarring and social challenges after burn injuries, it is critical for survivors to attend follow-up appointments to minimize adverse outcomes. Previous studies by our group have shown that follow-up after burn injuries is closely linked to social factors (such as drug dependence and housing insecurity), burn size, and distance from the clinic. Survivors living in rural areas often reside farther from clinics and present different burn characteristics compared to urban residents. This study aims to examine whether living in a rural community affects burn survivors’ ability to obtain follow-up care.

**Methods:**

After receiving IRB approval, a retrospective chart review was conducted using electronic medical records of adult patients over the age of 50 who were admitted to the burn center between January 2021 and December 2022. Collected data included burn injury details, housing insecurity status, substance use, and patients’ reported zip codes. Rural-Urban Commuting Area (RUCA) codes were assigned based on patients’ home zip codes. Exclusion criteria included non-burn injuries, in-hospital mortality, transfer to other hospitals, no scheduled outpatient follow-up, insurance-related barriers to follow-up, and prisoner status. Statistical analysis was conducted using SAS software (version 9.4), with results presented as medians (interquartile range).

**Results:**

A total of 354 patients (median age 61, IQR = 13) were analyzed, with 243 males (68.6%) and a median burn size of 5% TBSA (IQR 8). Of these, 46 patients (13%) were housing insecure, 92 (26%) tested positive for toxicology, and 88 patients (24.9%) lived in rural areas. Rural patients were more likely to be White (20.5% vs. 15%, p=0.03), have larger burns (6% TBSA, IQR 8.8 vs. 4.5% TBSA, IQR 7.5, p=0.02), and experience longer hospital stays (13.5 days, IQR 20.5 vs. 10.5 days, IQR 18). Of the analyzed patients, 128 (36.2%) did not attend any follow-up appointments. Living in a rural area was not associated with failure to attend follow-up (33% vs. 37%, p=0.47). However, patients who did not participate in follow-up were more likely to be housing insecure (71.7% vs. 30.9%, p< 0.0001), have a positive toxicology screen (48.9% vs. 31.7%, p=0.01), have shorter hospital stays (9 days, IQR 17.5 vs. 13 days, IQR 16), and undergo fewer operations (0, IQR 1 vs. 1, IQR 1, p=0.0001).

**Conclusions:**

A high percentage of burn survivors do not attend follow-up appointments post-injury. Key factors influencing this include housing insecurity, positive toxicology, shorter hospital stays, and fewer surgical interventions. Rural residence did not significantly impact follow-up appointment attendance.

**Applicability of Research to Practice:**

Both social and burn-related factors determine follow-up care after burn injuries but is not influenced by whether a patient resides in a rural community.

**Funding for the Study:**

N/A

